# Decision-Making Strategy for the Treatment of Rheumatoid Arthritis-Associated Interstitial Lung Disease (RA-ILD)

**DOI:** 10.3390/jcm10173806

**Published:** 2021-08-25

**Authors:** Hideaki Yamakawa, Takashi Ogura, Hideto Kameda, Tomoo Kishaba, Tae Iwasawa, Tamiko Takemura, Kazuyoshi Kuwano

**Affiliations:** 1 Department of Respiratory Medicine, Saitama Red Cross Hospital, 1-5 Shintoshin, Chuo-ku, Saitama 330-8553, Japan; 2 Department of Internal Medicine, Division of Respiratory Diseases, The Jikei University School of Medicine, 3-25-8 Nishi-Shinbashi, Minato-ku, Tokyo 105-8461, Japan; kkuwano@jikei.ac.jp; 3 Department of Respiratory Medicine, Kanagawa Cardiovascular and Respiratory Center, 6-16-1 Tomioka-higashi, Kanazawa-ku, Yokohama 236-0051, Japan; ogura@kanagawa-junko.jp; 4 Department of Internal Medicine, Division of Rheumatology, Faculty of Medicine, Toho University, 2-22-36 Ohashi, Meguro-ku, Tokyo 153-8515, Japan; hideto.kameda@med.toho-u.ac.jp; 5 Department of Respiratory Medicine, Okinawa Chubu Hospital, Okinawa, 81 Miyazato, Uruma 904-2293, Japan; kishabatomoo@gmail.com; 6 Department of Radiology, Kanagawa Cardiovascular and Respiratory Center, 6-16-1 Tomioka-higashi, Kanazawa-ku, Yokohama 236-0051, Japan; tae_i_md@wb3.so-net.ne.jp; 7 Department of Pathology, Kanagawa Cardiovascular and Respiratory Center, 6-16-1 Tomioka-higashi, Kanazawa-ku, Yokohama 236-0051, Japan; tamikobyori@gmail.com

**Keywords:** antifibrotic agents, DMARDs, interstitial lung disease, rheumatoid arthritis, therapy

## Abstract

Rheumatoid arthritis (RA) is a common type of autoimmune arthritis. Patient clinical outcomes might be influenced by numerous respiratory diseases, but interstitial lung disease (ILD) is the most important comorbidity. RA-associated ILD (RA-ILD) is divided into acute/subacute and chronic forms. In the acute/subacute course, if the disease is severe as indicated by a diffuse alveolar damage pattern, high-dose corticosteroids combined with antimicrobial agents should be promptly initiated while considering the differential diagnoses, primarily acute exacerbation (AE) of RA-ILD, drug-induced pneumonitis, and Pneumocystis pneumonia. As initial therapeutic management in the chronic course, the RA itself should be stabilized without delay; thereafter, the activity of ILD itself can be stabilized, considering the safety of each anti-rheumatic drug. The formation of the usual interstitial pneumonia (UIP) pattern is the most important determinant because lung function can worsen more quickly with this pattern. However, because clinicians can fail to identify specific radiological patterns, it is important to determine whether each patient with RA-ILD has UIP-like lesions such as subpleural reticulation, traction bronchiectasis, and honeycombing especially progressively enlarged cysts. In patients with progressive RA-ILD and high risk for infection or AE of ILD in whom fibrosis is dominant, clinicians should consider starting an anti-fibrotic agent.

## 1. Introduction

Rheumatoid arthritis (RA) is a systemic inflammatory disease with a prevalence of approximately 0.5–1.0% in the general population [1]. The prevalence of RA-associated interstitial lung disease (RA-ILD) ranges widely in the literature, likely due to varying methodologies used in its detection. Some studies identified RA-ILD through clinical detection, with prevalence estimates ranging from 2 to 8% in RA patients [2]. The most common reasons for death in RA patients in Japan are both respiratory disease and malignancy [3]. Among the respiratory diseases, ILD is the most predominant manifestation, and serious morbidity and increased mortality can be expected in some patients with RA-ILD [3,4]. Interestingly, a recent report showed that patients with RA-ILD are also at increased risk for cancer mortality [5]. Compared with RA without ILD, RA-ILD was clearly associated with poor prognosis [3,4,5]. Clinical physicians should thus recognize that the management of RA-ILD affects the prognosis of RA patients because ILD is a frequent manifestation and a poor prognostic factor in RA. The present review aimed to assess the characteristics of RA-ILD through an overview of previous studies in this field and then focus on the future treatment of RA-ILD.

## 2. Important Points in the Management of RA-ILD

Initially, clinicians should confirm whether the onset of RA-ILD is of the acute/subacute or chronic type, after which they can carefully discriminate among the wide variety of differential diagnoses. If patients present with an acute/subacute disease course, their life can be at risk even if the abnormalities on chest high-resolution computed tomography (HRCT) appear to be mild. In contrast, in patients with the chronic form of RA-ILD, clinicians should evaluate the activity of both arthritis and ILD and try to detect the early phase of an acute exacerbation (AE) of ILD, infection, and malignancy as soon as possible because these factors are associated with death in these patients [5].

## 3. Acute/Subacute Course of RA-ILD

This section may be divided into subheadings. It should provide a concise and precise description of the experimental results, their interpretation, as well as the experimental conclusions that can be drawn.

### 3.1. Diagnostic Assessment

The acute/subacute course of RA-ILD presents patterns of organizing pneumonia (OP) and diffuse alveolar damage (DAD). The OP pattern is suspected in cases refractory to antimicrobial drugs or that show wandering shadows, and almost all cases of this pattern resolve with a low to medium dose of corticosteroid therapy or even resolve spontaneously, which leads to a favorable prognosis [6].

When acute respiratory failure is accompanied by a wide range of pulmonary shadows radiographically, RA associated with diffuse alveolar damage (RA-DAD) needs to be distinguished from a variety of etiologies [6,7]. If the possibility of acute cardiac failure can be excluded, three major differential diagnoses are possible: pulmonary infection, RA-ILD itself (i.e., RA-DAD), and drug-induced pneumonia. If previous chest HRCT images are available, the clinician can confirm whether the patient has had RA-ILD from the beginning. Because most cases of RA-DAD are an AE of existing RA-ILD, RA-DAD is of low priority if the patient has no existing ILD [7]. Next to anticancer drugs, RA drugs such as methotrexate (MTX) and leflunomide can cause drug-induced pneumonitis, and Pneumocystis pneumonia (PCP) with RA is a well-known pulmonary infection that is sometimes encountered during treatment with RA drugs, particularly MTX and biologics [8,9,10,11,12]. For example, differentiating acute respiratory failure caused by MTX-induced pneumonitis from PCP and AE of RA-ILD is not always easy because these three clinical entities might present similar HRCT findings. Therefore, clinicians should consult the pharmaceutical information on RA drugs.

### 3.2. Therapeutic Assessment of Acute/Subacute RA-ILD Showing a Wide Variety of Bilateral Pulmonary Shadows

**Figure 1:**
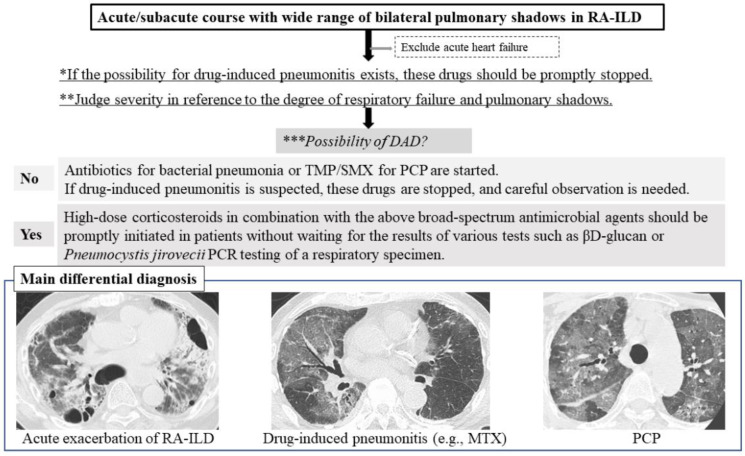
Three differential diagnoses of the acute/subacute course of RA-ILD are acute exacerbation of RA-ILD, drug-induced pneumonitis, and PCP. Abbreviations: DAD = diffuse alveolar damage; MTX = methotrexate; PCP = Pneumocystis pneumonia; RA-ILD = rheumatoid arthritis-associated interstitial lung disease; TMP/SMX = trimethoprim/sulfamethoxazole. Flow chart. First *, if the possibility of drug-induced pneumonitis exists, the drugs treating RA should be stopped promptly. Second, clinicians should judge severity ** and the possibility of DAD ***. Three differential diagnoses of the acute/subacute course of RA-ILD are acute exacerbation of RA-ILD, drug-induced pneumonitis, and PCP.

Several steps need to be taken in the assessment of acute/subacute RA-ILD. First, if the possibility of drug-induced pneumonitis exists, the drugs treating RA should be stopped promptly based on consideration of the balance between RA activity and respiratory status [13]. See the flow chart in Figure 1.

Second, the severity of pulmonary disease, which is indicated by the degree of respiratory failure and pulmonary shadows present and whether a DAD pattern is observed, needs to be assessed in each patient with RA-ILD. Considering that the pattern appears as mild pulmonary shadows in the patient in respiratory failure, a DAD pattern may be possible. In the radiological pattern of DAD shown by HRCT, heterogeneous foci of consolidation and ground-glass opacity (GGO) with a gravitationally dependent gradient are present along with increased consolidation in the posterobasal portions of the lungs [14]. A diffuse “crazy-paving” pattern can also be seen [14]. With organization and fibrosis, reticulation and traction bronchiectasis may develop, which often shows an anterior predominance.

Third, in cases with mild severity and an infectious clinical scenario, antibiotics to treat bacterial pneumonia and first-line therapy with trimethoprim/sulfamethoxazole (TMP/SMX) for PCP have likely already been started [15]. If drug-induced pneumonitis is suspected, causative drugs are stopped and careful observation is begun. However, in the case of high severity, as suggested by the presence of DAD, high-dose corticosteroids in combination with broad-spectrum antimicrobial agents including TMP/SMX should be promptly initiated without waiting for the results of tests such as βD-glucan or PCR for *Pneumocystis jirovecii* in respiratory specimens [15]. Although the efficacy of this therapy is uncertain in the case of AE of RA-ILD, severe PCP and drug-induced pneumonitis such as that induced by MTX may be improved by high-dose corticosteroid administration [15,16]. If the response to corticosteroid therapy is poor, the administration of other immunosuppressive agents such as cyclophosphamide (CYC), cyclosporine A, and tacrolimus should be considered, which has the potential to improve the prognosis of the AE of RA-ILD [17]. The presence of AE of RA-ILD indicates an extremely poor condition with high mortality (64%) [2,4,5], and thus, this serious condition needs to be carefully considered.

## 4. Chronic Course of RA-ILD

### 4.1. Prevalence and Clinical Manifestations

A significant percentage of patients have ILD either predating (10%) or concomitant with (17%) the diagnosis of RA. About half of the patients develop ILD either before or within 5 years of the RA diagnosis [18]. Some studies identified prevalence estimates ranging from 2 to 8% in RA patients [2], whereas the estimated prevalence based on HRCT analysis is reported at a relatively higher range of 27–67% [19,20,21,22,23,24,25,26,27]. Most cases are diagnosed as being asymptomatic, with 7–29% having clinical symptoms [24,25,26,27]. Although the true prevalence of ILD in RA has not been established, importantly, clinically evident ILD may occur in approximately 10% of RA patients [19,20,21,22,23,24,25,26,27,28].

### 4.2. Disease Behavior

In 2017, Zamora-Legoff et al. reported that over a 5-year period after the diagnosis of RA-ILD (3.3-year median follow-up time from ILD diagnosis), 40% of patients developed a diffusing capacity for carbon monoxide (DLCO) of <40% predicted and 22% developed a forced vital capacity (FVC) of <50% predicted, which indicated severe restrictive disorder, and a third of the patients required supplemental oxygen [29]. Of the patients without respiratory restriction at the initial diagnosis, about 10% of them had developed severe restrictive disorder over the 5 years [29]. Mena-Vázquez et al. reported in 2021 that 19.8% of RA-ILD patients had experienced progression (worsening of FVC of >10% or DLCO of >15% and radiological progression) over the 5 years from their ILD diagnosis [30]. These patients were classified as having progressive fibrosing ILD (PF-ILD) [31,32]. Although presently there are only a few reports of ILD disease progression, which vary based on the detection methods used, taken together, at least 10% of RA-ILD patients might experience progression over the long-term course of the disease.

### 4.3. Predictors of ILD Progression and Prognostic Factors

The independent predictors of worsening of RA-ILD were reported to be the usual interstitial pneumonia (UIP) pattern, lowered FVC (e.g., <80%), cigarette smoking, and higher anticitrullinated protein antibody titers [20,25,30,33]. Mena-Vázquez et al. reported that treatment with abatacept (ABT) as a synthetic analog of CTLA-4Ig, tocilizumab (TCZ) as an interleukin-6 inhibitor (IL-6i), and rituximab (RTX) as an anti-CD20 chimeric antibody was associated with predictors of stabilized pulmonary function [30]. Factors significantly related to mortality in RA were male sex, older age, having ILD, and higher articular activity [5]. Poor prognostic factors in RA-ILD include male sex, older age, lower FVC and DLCO, deterioration in FVC and DLco over 6 months, wide areas of pulmonary fibrosis, UIP pattern, and honeycombing [4,7,34,35,36,37,38].

### 4.4. Prognosis and Causes of Death Compared with Idiopathic Pulmonary Fibrosis

**Figure 2:**
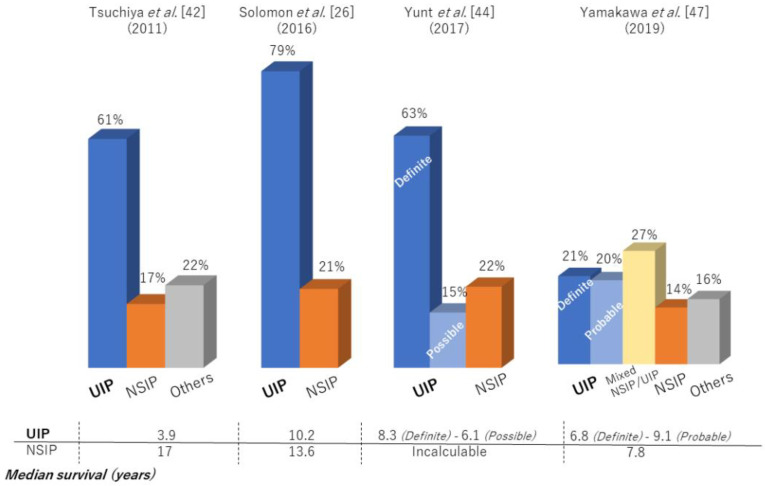
High-resolution computed tomography patterns and median survival of patients with RA-ILD.

**Figure 3:**
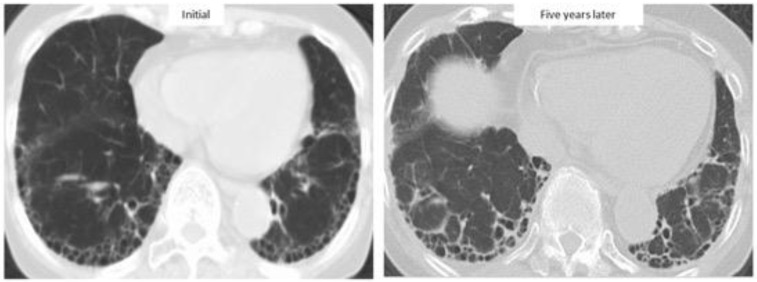
High-resolution computed tomographic images of a patient with a stable course of RA-ILD for five years.

Previous large cohort studies reported that median survival after the diagnosis of RA-ILD was only 2.6–3.0 years with a 5-year survival rate of 35–39%, similar to that for idiopathic pulmonary fibrosis (IPF) [4,39]. In fact, RA-ILD has also been reported to be associated with IPF-related genes: MUC5B promoter variant and leukocyte telomere length [40,41]. In particular, the prognosis of RA-ILD patients with a UIP pattern, as with that of IPF, was poorer than the prognosis of patients with a non-UIP pattern [4,7,22,28]. However, prognosis in RA-ILD varied across the reports (median survival for RA-UIP of 3.2–10.2 years and RA-nonspecific interstitial pneumonia [NSIP] of 7.8–17 years) (Table 1, Figure 2), with recent reports showing that survival for RA-ILD with a UIP pattern was significantly better than that for IPF [37,42,43,44,45,46,47]. Figure 3 depicts a case with a stable course of RA-UIP with honeycombing in which the fibrotic area does not really deteriorate over 5 years. The reason for the different results regarding prognosis may be explained by the following three points.

1: Sample bias in the diagnosis of RA-ILD, as this depends on whether a respiratory or rheumatology physician made the diagnosis. Many patients were diagnosed by rheumatologists as not having respiratory symptoms, whereas most respiratory physicians often find that RA patients have progressive ILD and/or respiratory symptoms in clinical practice [26,42,43,47].

**Figure 4:**
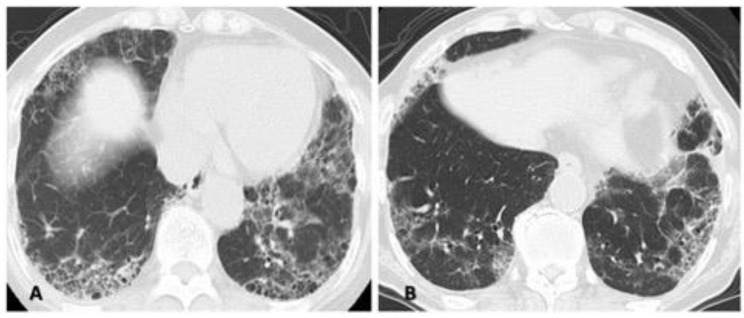
Two cases illustrating the difficulty of classifying a specific high-resolution computed tomography pattern as a mixed NSIP/UIP pattern. Both central or diffuse distribution of reticulation or ground-glass opacity as the component of NSIP and subpleural reticulations with or without honeycombing as the component of UIP in the lower lung are shown. (**A**) A 71-year-old woman and (**B**) a 72-year-old man. Abbreviations: NSIP = nonspecific interstitial pneumonia; UIP = usual interstitial pneumonia.

2: Differences in the methods of analyzing or in the analysts who assess the HRCT pattern. In accordance with the times, classification guidelines for idiopathic interstitial pneumonia and IPF are often used, and most patients are classified as having different subtypes (e.g., UIP, NSIP) based on HRCT. However, an overlapping or indeterminate pattern (i.e., an unclassifiable pattern other than UIP, NSIP, or OP) is present to some extent in 6 to 52% of the RA-ILD population because connective tissue disease-associated ILD presents a diversity of patterns [23,47,48,49]. Therefore, we often cannot classify an HRCT pattern specifically as either UIP or NSIP in clinical practice as shown in Figure 4. Thus, the correlation between HRCT pattern and prognosis can vary.

**Figure 5:**
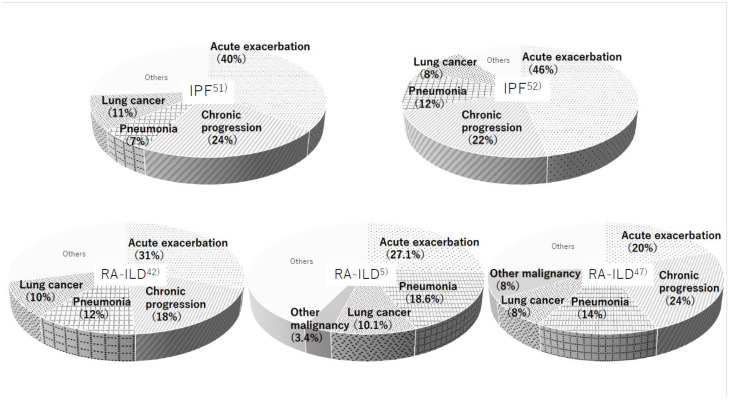
Causes of death were reported by five different studies in patients with idiopathic pulmonary fibrosis (IPF) (**upper row**) and RA-ILD (**lower row**).

3: Infection including pneumonia is the more frequent cause of death rather than IPF. The most common causes of death in RA patients were malignancy (24%), cardiovascular disorder (14%), infectious pneumonia (12%), and ILD (11%) as reported by Nakajima et al. [50]. Regardless of whether the etiology was IPF or RA-ILD, three major reasons for the cause of death were AE of ILD (IPF: 40–46%, RA-ILD: 20–31%), chronic progression (IPF: 22–24%, RA-ILD: 18–24%), and lung cancer (IPF: 8–11%, RA-ILD: 8–10%) (Figure 5) [3,5,42,47,50,51,52]. The frequency of infectious pneumonia as a cause of death is somewhat higher in RA-ILD versus IPF (12–18.6% vs. 7–12%). Patients with RA have elevated susceptibility to serious infections due to features of the disease itself, comorbidities, and immunosuppressive treatment [2,5,28]. In other words, the prognosis in patients with RA-ILD may improve if clinicians can control any infectious complications well.

**Figure 6:**
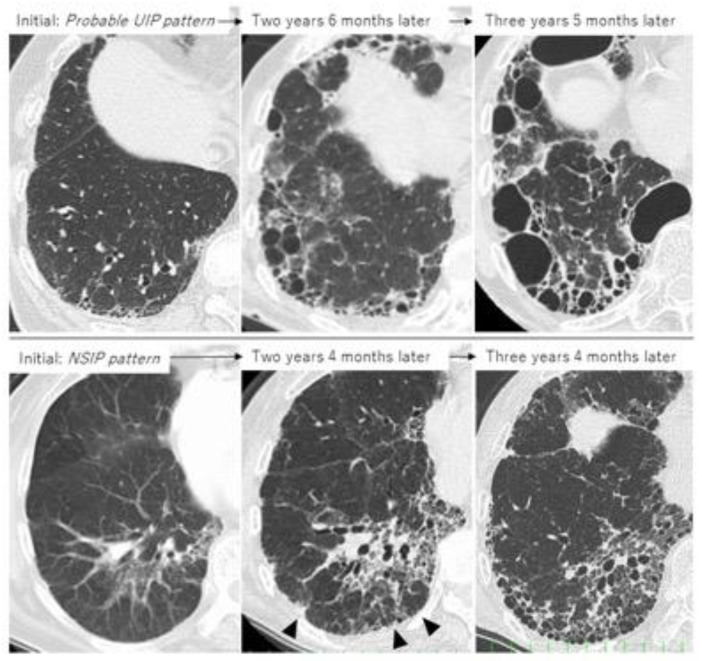
Two cases of progressive RA-ILD showing the evolution of honeycombing during follow-up. Upper row: probable UIP pattern at initial imaging; lower row: NSIP pattern at initial imaging (arrowhead indicates UIP-like lesion formation as subpleural reticulation).

Taken together, the first important thing when determining treatment is not only to clearly judge whether the patient shows a UIP pattern or non-UIP pattern, such as an NSIP pattern, but also whether each patient with RA-ILD has UIP-like lesions (i.e., subpleural reticulation with traction bronchiectasis, honeycombing) because HRCT can often fail to correctly identify patterns such as UIP, NSIP, and unclassifiable [53]. Although not all RA-UIP shows a progressive course, clinicians should consider the possibility that patients will develop progressive ILD if they have UIP-like lesions at the time of diagnosis (Figure 6, upper row) [53]. Of note, even though the initial HRCT pattern might be one of NSIP, UIP-like lesions can become apparent and then progress to ILD (Figure 6, lower row) [36,53,54]. The second important thing in the management of RA-ILD is the prevention and early detection of infectious complications including pneumonia [3,5,28]. In addition, for RA-ILD patients to have a better prognosis than that for IPF, early detection of malignancy may be essential for those who are male or smokers [5].

### 4.5. Therapeutic Assessment of the Chronic Course of RA-ILD

**Figure 7:**
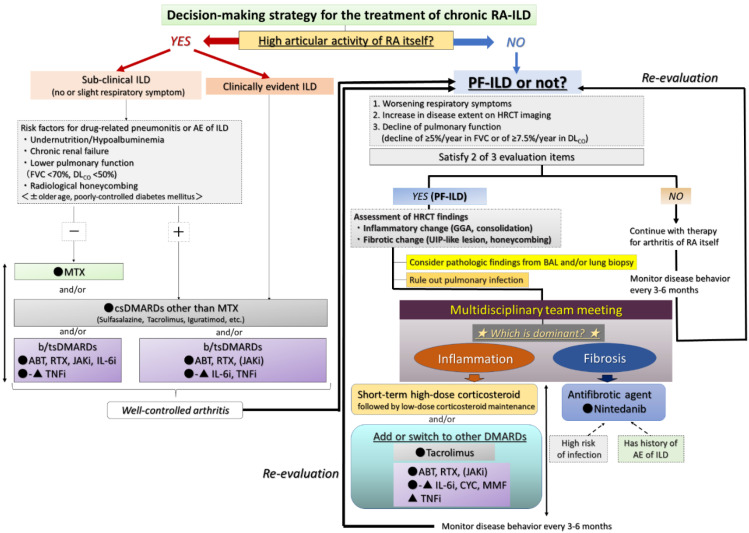
Proposed management algorithm for chronic RA-ILD. Abbreviations: ABT = abatacept; AE = acute exacerbation; BAL = bronchoalveolar lavage; b/tsDMARD = biologic/targeted synthetic DMARD; csDMARD = conventional synthetic DMARD; DLCO = diffusing capacity for carbon monoxide; DMARD = disease-modifying anti-rheumatic drug; FVC = forced vital capacity; GGA = ground-glass attenuation; ILD = interstitial lung disease; JAKi = Janus kinase inhibitor; MTX = methotrexate; PF-ILD = progressive fibrosing ILD; RA = rheumatoid arthritis; RTX = rituximab; IL-6i = interluekin-6 inhibitor; CYC = cyclophosphamide; MMF = mycophenolate mofetil; TNFi = tumor necrosis factor-inhibitor; UIP = usual interstitial pneumonia.

The therapeutic assessment of RA-ILD is difficult due to the lack of evidence and can be controversial [2,46,52]. Two main factors are important in the decision to start treatment: judging whether A) arthritis itself is controllable and B) the patient has progressive ILD (see the flow chart in Figure 7) [55,56,57]. Although the judgment of the latter can be difficult, the concept and definition of PF-ILD, which includes RA-ILD, were created in the INBUILD trial and are also useful in clinical practice [31]. The definition is based on the progression of fibrosis as assessed by FVC and HRCT findings and worsening of respiratory symptoms at a certain rate or even faster [31]. Based on this definition, Cottin et al. proposed that the criteria for PF-ILD include a relative decline of ≥10% in FVC; a relative decline of ≥15% in DLCO; or worsening symptoms or a worsening radiological appearance accompanied by a ≥5 to <10% relative decrease in FVC within 24 months [32]. Moreover, we also proposed that progressive ILD be defined as a disease showing a relative decline in FVC of ≥10%/year and/or that in DLCO of ≥7.5%/year, which may be useful in clinical decision-making when determining therapy for fibrotic NSIP [58]. We present a therapeutic strategy for RA-ILD based on this concept of PF-ILD as below.

#### 4.5.1. Presence of High Articular Activity of RA Itself Regardless of ILD Activity

The arthritis activity of RA itself is one of the predictive factors of the development of RA-ILD [55]. In addition, higher activity of arthritis may be associated with the exacerbation of RA-ILD [56], and this can be a poor prognostic factor for patients with RA [5]. Therefore, if patients with RA-ILD suffer from symptoms of arthritis, arthritis should be treated first and then stabilized. Then, it can be determined whether the RA-ILD is sub-clinical ILD or clinically evident ILD (Figure 7).

Although the association between MTX and the pulmonary toxicity of ILD has been reported for some time, a recent report showed MTX use not to be associated with an increased risk of RA-ILD in patients with RA and that ILD was detected later in MTX-treated patients [59]. As one of the disease-modifying anti-rheumatic drugs (DMARDs), MTX is one of the most effective medications available to treat RA [60,61]. Thus, RA patients with sub-clinical ILD and no risk factors for drug-related pneumonitis or AE of ILD (e.g., undernutrition, chronic renal failure, reduced pulmonary function, or radiological honeycombing) should be treated with MTX without delay [55]. If the patient has any of the above risk factors or clinically evident ILD, the use of conventional synthetic DMARDs (csDMARDs) other than MTX (e.g., sulfasalazine, tacrolimus, or iguratimod) and/or biologic/targeted synthetic DMARDs (b/tsDMARD) should be considered [62]. In regard to the csDMARD leflunomide, because pre-existing ILD was the most important risk factor for the occurrence of leflunomide-induced ILD, we suggest that leflunomide not be prescribed for RA patients complicated by ILD [63]. Other csDMARDs such as sulfasalazine and tacrolimus are thought to provide some safety for the lung [57].

The safety of bDMARDs and tsDMARD and their effect on RA-ILD are not clear [62]. Although the possible effectiveness of bDMARDs in RA-ILD has been described in only a few retrospective studies and anecdotally, almost all of the bDMARDs have the possibility of improving and/or stabilizing lung function [57]. Therefore, therapeutic management should emphasize safety in regard to ILD [62]. Among the bDMARDs, ABT, TCZ, and RTX seem to be relatively safe for use in RA-ILD [55,57,62,64]. However, care must be taken with TCZ because some reports have shown it to be associated with the exacerbation of pre-existing RA-ILD, although TCZ demonstrated a potential effect on the stabilization of RA-ILD [65,66], and it prevents the progression of early systemic sclerosis-associated ILD [67]. Therefore, TCZ as an IL-6i may be effective for early or mild RA-ILD but should be avoided for clinically evident ILD or PF-ILD. Sarilumab is another IL-6i, but currently, there is no data on its efficacy in RA-ILD. In contrast, a recent review showed increased mortality with the use of tumor necrosis factor inhibitors (TNFi) in RA-ILD; therefore, TNFi may have lower priority in the treatment of RA-ILD [55,68]. Janus-kinase inhibitor (JAKi) as a tsDMARD was reported to cause a very low rate of ILD exacerbation [69,70]. Moreover, several reports have recently described the effectiveness of JAKi in fatal anti-melanoma differentiation-associated protein 5 antibody-positive RP-ILD [71,72]. Although we cannot state this definitively, JAKi may provide additional benefit in the therapy of RA-ILD that is not limited just to that of the arthritis of RA. Taken together, clinicians should administer different DMARDs depending on their safety in RA-ILD and first stabilize the arthritis of RA itself without delay. Thereafter, the activity of ILD itself can become the treatment focus.

#### 4.5.2. Presence of Progressive ILD Itself despite Stable Arthritis

As mentioned above, we judge whether the patient with RA-ILD has PF-ILD based on respiratory symptoms, disease extent on HRCT, and pulmonary function [31,32,58]. Even if the patients are thought not to have PF-ILD, their disease behavior is monitored every 3 to 6 months, and then we re-evaluate each time whether the patient has progressed to PF-ILD [73] (Figure 7). Needless to say, a multidisciplinary team meeting (including pulmonologist, rheumatologist, radiologist, and pathologist) is the preferred method for assessment for RA-ILD.

**Figure 8:**
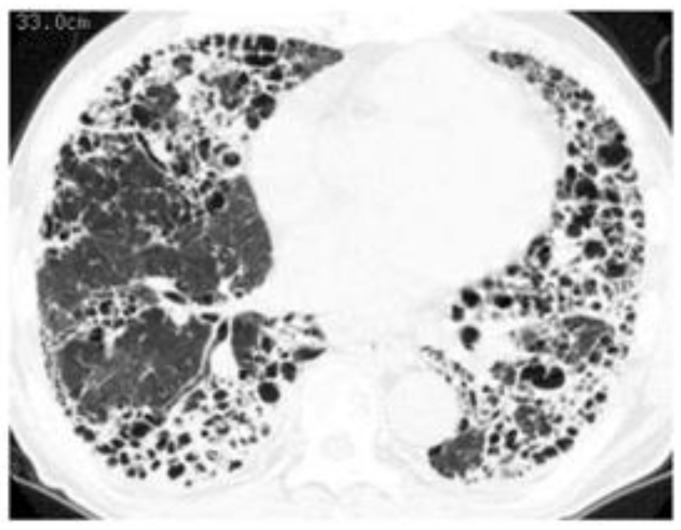
A case of RA-ILD in which the complication of nontuberculous mycobacterial pulmonary disease (*Mycobacterium kansasii* infection) developed. Chest computed tomography showed consolidation along with honeycombing.

**Figure 9:**
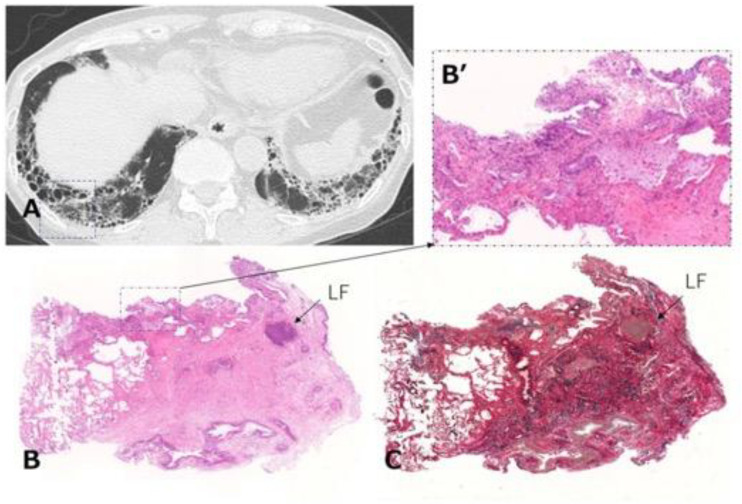
Transbronchial lung cryobiopsy in a case of RA-ILD (73-year-old woman). (**A**) Chest CT shows subpleural reticulations with honeycombing as the component of UIP in the lower lung. Lung cryobiopsy was performed in the right B9 (dashed square). (**B**) Hematoxylin and eosin staining show the lesion to be characterized by dense collagenous fibrosis with architectural destruction as a UIP pattern along with inflammatory cells including LF in the interstitial tissue. (**B’**) A high-power magnification view of the dashed square in (**B**) shows a mild inflammatory change. (**C**) Elastica van Gieson staining shows the lesion as in (**B**). Abbreviations: RA = rheumatoid arthritis; ILD = interstitial lung disease; CT = computed tomography; UIP = usual interstitial pneumonia; LF = lymphoid follicle.

When we determine that a patient with RA-ILD has PF-ILD, we focus on the assessment of inflammatory or fibrotic changes in the HRCT findings. Inflammatory change is assumed when GGO and consolidation are present. As a caveat, GGO, which is a finding typically associated with inflammation or infiltration, does not always represent reversible lung disease, but rather in some cases, it may represent microscopic fibrosis [74], whereas fibrotic change is included in reticulation with traction bronchiectasis and honeycombing [36,38,53]. However, all etiologies associated with ILD can lead to a poor prognosis, particularly in cases in which a component of UIP is found to a certain degree [53,58,73]. In other words, some cases represent the natural evolution of the inflammatory change of ILD into a UIP pattern indicating fibrotic change [38,75]. In fact, we reported that a certain number of patients with RA-ILD developed honeycombing during long-term follow-up, regardless of whether they had UIP or NSIP, and that their median survival time was poor at 3.2 years after the formation of honeycombing [38]. Therefore, of importance when judging fibrotic change is whether UIP-like lesions (i.e., predominantly subpleural reticulation with traction bronchiectasis) and honeycombing have formed during the follow-up period. Although HRCT is considered the best tool in the evaluation of ILD, bronchoalveolar lavage and transbronchial lung biopsy may also be useful only when the possibility of combined pulmonary infection exists (Figure 8) or when it is difficult to judge which is the dominant condition, inflammation or fibrosis (Figure 9) [76]. However, because surgical lung biopsy was reportedly involved in a high rate of in-hospital deaths, it should be discouraged [77].

**Figure 10:**
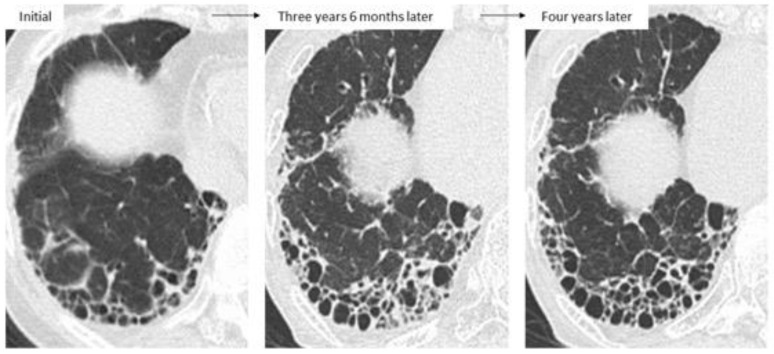
A patient who received sulfasalazine monotherapy for RA. Afterward, UIP-like lesions appearing as reticulations and honeycombing extended with worsening of the patient’s respiratory symptoms, and wall thickening in the areas of honeycombing were also present at 3 years and 6 months after the initial image. After two courses of pulse dose methylprednisolone followed by low-dose prednisolone, wall thickening had improved by six months later.

If we judge that inflammation is dominant in PF-ILD, we augment the basic RA therapy by strengthening the anti-inflammatory therapy. When administering anti-inflammatory therapy, we consider the use of a corticosteroid and/or adding or switching to other DMARDs. Yamano et al. reported that short-term high-dose corticosteroid followed by low-dose maintenance with tacrolimus significantly improved connective tissue disease-associated ILD including RA-ILD [78]. Some reports showed that corticosteroid improved or stabilized pulmonary function even if a UIP pattern was present [22,37,78]. As already mentioned, classification of specific HRCT patterns is often difficult, and RA-UIP frequently shows inflammatory pathologic change appearing as lymphoid hyperplasia unlike with IPF/UIP [23,47,49]. An example is shown in Figure 10, in which the patient had UIP-like lesions (i.e., predominant subpleural reticulation and honeycombing) indicative of RA-UIP. However, wall thickening of honeycombing resolved after high-dose corticosteroid therapy. Nevertheless, prednisolone doses higher than 10 mg are associated with a high risk of infectious comorbidity [79]. Therefore, treatment with short-term high-dose corticosteroids followed by low-dose maintenance may be one effective therapy for RA-ILD including UIP. The efficacy of tacrolimus was also reported in myositis-associated ILD in addition to RA-ILD, and thus it might be another useful option [78,80]. As mentioned in the previous section, when keeping the safety of b/tsDMARDs in mind, the first priority in treatment is ABT or RTX, followed by JAKi or TCZ and a TNFi. CYC and mycophenolate mofetil have a modest effect on systemic sclerosis-associated ILD [81,82], but there is little data on these drugs that indicate an effect on the clinical improvement and stability of RA-ILD [45,83].

**Figure 11:**
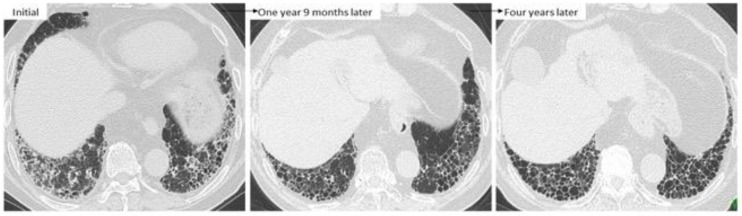
A case of RA-ILD showing ground-glass opacity around reticulations. This finding was improved at one year 9 months after treatment with anti-inflammatory agents such as tacrolimus and TNF inhibitor. However, UIP-like lesions (i.e., predominantly subpleural reticulation and honeycombing) without inflammatory change were obvious four years later.

If we determine that fibrosis is dominant in PF-ILD, we consider starting an antifibrotic agent such as nintedanib, which has been approved for the treatment of IPF in more than 70 countries and is effective and well-tolerated for the treatment of IPF [84,85]. Recently, the INBUILD study showed the effectiveness of nintedanib not only for IPF but PF-ILD including RA-ILD [31]. In patients who present with UIP-like lesions such as predominant subpleural reticulation and honeycombing seen on HRCT, lung function is known to worsen more quickly than that in patients presenting other patterns [53]. As shown in Figure 11, GGO around reticulations can improve with the administration of the anti-inflammatory agent for arthritis itself, and after that, the UIP-like lesions become obvious along with the formation of honeycombing. Because prognosis after the formation of honeycombing is poor [38], if UIP-like lesions become obvious without inflammatory changes as indicated by GGO and wall thickening of honeycombing, early initiation of nintedanib should be considered. Interestingly, nintedanib might have the potential to suppress the AE of ILD [31], which is a most harmful event and can even be fatal [7,28,47]. Therefore, nintedanib is expected to be advantageous in the treatment of RA-ILD, and for patients with a history of AE of RA-ILD, priority intervention with nintedanib should be considered to help halt the progression to PF-ILD in RA. Moreover, particularly in cases in which it is difficult to judge whether inflammation or fibrosis is the dominant condition if a patient has factors predictive of a high risk of infection, nintedanib may be preferable to anti-inflammatory agents. Importantly, even though therapy such as an anti-inflammatory or anti-fibrotic agents is being administered, disease behavior should be monitored every 3–6 months to re-evaluate whether the RA-ILD remains under control.

## 5. Conclusions

RA-ILD can be divided into acute/subacute and chronic forms when considering therapeutic strategy. In the acute/subacute form, if the disease is severe as suggested by a DAD pattern, high-dose corticosteroids in combination with antimicrobial agents should be promptly initiated while considering the differential diagnosis, which is mainly AE of RA-ILD, drug-induced pneumonitis, or *Pneumocystis* pneumonia. In the chronic form of RA-ILD, the arthritis of RA itself should first be stabilized without delay, and afterward, the activity of ILD itself can be stabilized, considering the safety of each anti-rheumatic drug for use in RA-ILD. The formation or presence of UIP-like lesions can be related to progressive ILD, although HRCT can fail to identify specific patterns in actual practice. If inflammatory change is more dominant than fibrotic change, anti-inflammatory therapy should be strengthened. If fibrosis is dominant in patients with progressive RA-ILD and a high risk of infection or AE of ILD, clinicians should consider starting an antifibrotic agent. Prioritization of anti-inflammatory therapies or antifibrotic therapies will presumably remain an issue that must be solved in the context of individual diseases [53]. Thus, analysis of the HRCT findings is pivotal, and multidisciplinary discussion that includes the pulmonologist, rheumatologist, radiologist, and pathologist is needed to standardize the decision-making strategy for treatment that is necessary to correctly approach the diagnosis of patients with RA-ILD.

## Figures and Tables

**Table 1:** HRCT patterns and prognosis of RA-ILD.

Authors	Year	No. of Patients	HRCT Patterns	Reference to Criteria of HRCT Classification	5-Year Survival Rate (%)	Median Survival
Kim et al. [23]	2010	82	24% (UIP) 23% (NSIP) 51% (Indeterminate) 2% (Others)	*2002 ATS/ERS classification of the IIPs*	N/A	3.2 years (UIP) 6.6 years (Non-UIP)
Tsuchiya et al. [42]	2011	94	61% (UIP) 17% (NSIP) 22% (Others)	*2002 ATS/ERS classification of the IIPs*	36.6% (UIP) 93.8% (NSIP)	3.9 years (UIP) 17 years (NSIP)
Kelly et al. [25]	2014	230	65% (UIP) 24% (NSIP) 11% (Others)	N/A	N/A	N/A
Solomon et al. [26]	2016	137	79% (Definite + Possible UIP) 21% (NSIP)	*2011 IPF guideline*	N/A	10.2 years (Definite+Possible UIP) 13.6 years (NSIP)
Morisset et al. [43]	2017	309	24% (Definite UIP) 16% (Possible UIP) 60% (Inconsistent with UIP)	*2011 IPF guideline*	N/A	N/A
Zamora-Legoff et al. [20]	2017	181	54% (UIP) 40% (NSIP) 6% (OP)	N/A	55.2% (UIP) 65.0% (NSIP) 47.4% (OP)	N/A
Yunt et al. [44]	2017	158	63% (Definite UIP) 15% (Possible UIP) 22% (NSIP)	*2011 IPF guideline*	N/A	8.3 years (Definite UIP) 6.1 years (Possible UIP) Incalculable (NSIP)
Fu et al. [45]	2019	266	17% (Definite UIP) 20% (Possible UIP) 32% (Unclassifiable) 30% (Others)	*2011 IPF guideline*	69.7% (All)	N/A
Jacob et al. [46]	2019	157	35% (Definite UIP) 36% (Probable UIP) 29% (Inconsistent with UIP)	*2018 Diagnostic criteria for IPF:* *Fleischner Society*	<6-year survival rate > 45% (Definite UIP) 58% (Probable UIP)	N/A
Yamakawa et al. [47]	2019	112	21% (Definite UIP) 20% (Probable UIP) 27% (Mixed NSIP/UIP) 29% (Alternative)	*2018 IPF guideline*	70.2% (Definite UIP) 90.9% (Probable UIP) 80.0% (Mixed NSIP/UIP) 90.0% (NSIP)	6.8 years (Definite UIP) 9.1 years (Probable UIP) 8.6 years (Mixed NSIP/UIP) 7.8 years (NSIP)
Kakutani et al. [5]	2020	261	46% (Definite + Possible UIP) 54% (Non-UIP)	*2011 IPF guideline*	N/A	N/A

Abbreviations: ATS = American Thoracic Society; ERS = European Respiratory Society; HRCT = high-resolution computed tomography; IPF = idiopathic pulmonary fibrosis; NSIP = nonspecific interstitial pneumonia; OP = organizing pneumonia; RA-ILD = rheumatoid arthritis-associated interstitial lung disease; UIP = usual interstitial pneumonia.

## Data Availability

Not applicable.
